# The landscape of artificial intelligence-enabled medical devices in the EU and the US intended for intensive care units

**DOI:** 10.1038/s41746-026-02609-2

**Published:** 2026-04-10

**Authors:** Oscar Freyer, Stephan Buch, Adel Bassily-Marcus, Sven Zenker, Brian W. Pickering, Max Ostermann, Anett Schönfelder, Stephen Gilbert

**Affiliations:** 1 Else Kröner Fresenius Center for Digital Health, TUD Dresden University of Technology, Dresden, Germany; 2 Yale School of Medicine, Department of Surgery, Yale New Haven Health System, New Haven, CT USA; 3 Staff Unit for Medical & Scientific Technology Development & Coordination (MWTek), University Hospital Bonn, Bonn, Germany; 4 Department of Anesthesiology and Intensive Care Medicine, University Hospital Bonn, Bonn, Germany; 5 Institute for Medical Biometry, Informatics, and Epidemiology, University Hospital Bonn, Bonn, Germany; 6 Department of Anesthesiology and Perioperative Medicine, Mayo Clinic, Rochester, NY USA; 7 Faculty of Business and Economics, TUD Dresden University of Technology, Dresden, Germany

**Keywords:** Computational biology and bioinformatics, Engineering, Health care, Mathematics and computing, Medical research

## Abstract

Artificial intelligence (AI) is increasingly used in healthcare, yet translation into routine intensive care units (ICU) practice remains slow. Through a multimethod search, we identified 36 on-market ICU-specific AI-enabled medical devices in the US and EU, challenging previous research findings. Most devices focus on prediction. However, as availability does not equal proven benefit, adoption will depend less on the availability of additional models and more on addressing persistent implementation barriers.

## Introduction

Clinical research on artificial intelligence (AI) in healthcare has grown rapidly, covering traditional AI technologies, such as machine learning, as well as the latest innovations, such as Large Language Models (LLMs)^1^. In intensive and critical care, research focuses on diagnostic capabilities^2^, disease prediction^3^, therapy^4^, as well as ICU administration, e.g., for optimal resource allocation^5^, discharge planning^6^, and generation of discharge letters^5^.

Although this growing body of research is driven by the goal of improving patient care, its clinical relevance relies on its integration into routine ICU practice. This integration is often described as slow. According to Berkhout et al.^7^, AI models for ICU use cases often remain in early development or initial clinical validation stages, with only a few progressing to clinical integration and none reaching full implementation. In contrast, Lee et al.^8^ identified a low number of AI-enabled medical devices (AIeMD) available on the US market specifically designed for ICU use^8^, while other researchers describe the use of such commercial products in studies^9^.

This discrepancy between academic research-focused reports on translation and availability of authorized devices highlights an ambiguity in the current evidence base: it remains unclear which ICU-focused AIeMD (ICU-AIeMD) have moved from the research stage to the market. At the same time, there is a gap between regulatory authorization and proof of clinical utility, leading to limited integration into clinical workflows and slowing widespread adoption^10^. Additionally, existing analyses of AIeMD primarily focus on the US, supported by the availability of FDA documentation and curated device lists, whereas a comparable EU data foundation remains lacking^11^. This gap, however, is relevant, as the EU and US apply structurally different regulatory frameworks, including different risk classification schemes, conformity assessment routes, and transparency requirements^12^. A detailed overview of the regulatory context is provided in the Supplementary Material. These regulatory differences may shape both the types of devices that reach the market and the evidence publicly available about them. Therefore, this study aims to (1) systematically identify commercially available ICU-AIeMD in the US and the EU, and (2) characterize and compare their regulatory and functional features to support understanding of the translation from research into authorized products across both regions.

**Fig. 1:**
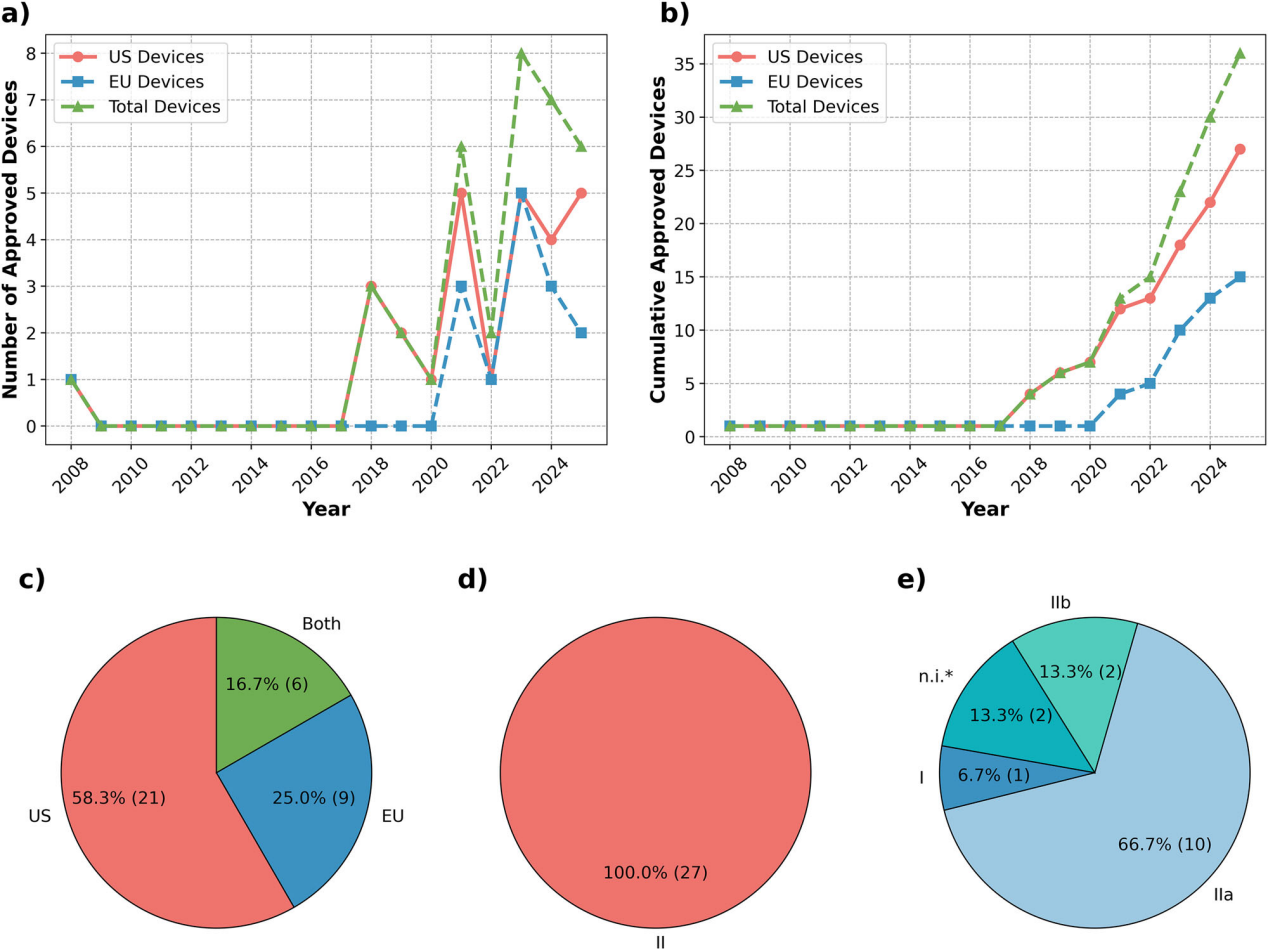
Regulatory aspects. **a** Number of approved devices by year, **b** cumulative number of approved devices, **c** market distribution, **d** risk class distribution of devices on the US market, **e** risk class distribution of devices on the EU market.

In our search, we have identified 36 on-market AIeMD intended for use in the ICU setting. A total of 25% (*n* = 9) are available in the EU (one of which is under an US Food and Drug Administration (FDA) Breakthrough Device Designation in the US and thus not officially approved), 58.3% (*n* = 21) in the US, and 16.7% (*n* = 6) in both regions (Table 1 and Fig. 1c). The first ICU device, Visensia, was approved in the US in 2008 and is available in both markets (Fig. 1a). Following this initial authorization, there was a 10-year gap, with authorizations resuming in 2018 in the US and in 2021 in the EU (Fig. 1a, b). All devices in the US (*n* = 27) fall into risk class II (Fig. 1d). There is a wider distribution in the EU, ranging from Class I to Class IIb (Fig. 1e).

**Table 1:** Devices intended for ICU settings

Name	Decision number/submission number/UDI	Year of first authorization	Source	US	Risk class	EU	Risk class	Intended use/description (based on FDA summary and manufacturer's website)	Disease/condition
Acumen Hypotension Prediction Index (HPI) Algorithm	K230057	2023	Lee et al.^8^	✓	II			Provides clinicians with insights into the likelihood of future hypotensive events and associated hemodynamics in surgical and non-surgical patients receiving advanced hemodynamic monitoring.	Hypotension
AHI System	K212219	2021	FDA Database	✓	II			Assists clinicians in hospital settings by using ECG analysis to identify or predict hemodynamic instability in adults.	Hemodynamic Instability
AI-ECG Platform	K180432	2018	Lee et al.^8^	✓	II			Provides AI-assisted analysis of 12-lead resting ECGs for the detection of common cardiac abnormalities in adults.	Cardiovascular Conditions
AI-ECG Tracker	K200036	2020	Lee et al.^8^	✓	II			Provides AI-assisted analysis of 12-lead ECG data from adults without pacemakers for the detection and assessment of arrhythmias	Cardiovascular Conditions
aiOS	UDI 07290018052198	2023^a^	Websearch			✓	I (MDR)	Provides a clinical AI platform that enables healthcare systems to securely manage, integrate, and scale multiple AI solutions with automated orchestration, built-in governance, and configurable workflows.	Administration
Airmod	K242798	2024	FDA Database	✓	II			Supports continuous, non-invasive monitoring of respiratory rate in sedated or anesthetized adult patients using acoustic signal analysis	Sedation Monitoring
Analytic for Hemodynamic Instability (AHI)	DEN200022	2021	FDA Database	✓	II			Analyses Lead-II ECG signals to identify patients who are showing signs of hemodynamic instability.	Hemodynamic Instability
BIAlert Sepsis	UDI 843702601003	2024	Websearch			✓	IIa (MDR)	Provides real-time intelligent alerts to support early detection and prediction of sepsis and septic shock in hospitalized patients using AI-driven analysis of clinical data.	Sepsis
BriefCase	K221330	2022	FDA Database	✓	II			Analyzes chest X-rays of patients aged 18 and older to flag suspected malposition of endotracheal tubes relative to the carina.	Vessel Occlusions
CLEWICU System	K233216	2024	FDA Database	✓	II			Provides clinicians in critical care settings with predictive insights into the likelihood of future hemodynamic instability and identifies adult patients with low risk of deterioration.	Hemodynamic Instability, Clinical Deterioration
CLEWICU System (ClewICUServer and ClewICUnitor)	K200717	2021	FDA Database	✓	II			Provides clinicians in critical care settings with predictive insights into the likelihood of future hemodynamic instability and identifies adult patients with low risk of deterioration.	Hemodynamic Instability, Clinical Deterioration
Critical Care Suite with Endotracheal Tube Positioning AI algorithm	K211161	2021	Websearch	✓	II	✓	IIa (MDD)	Analyzes frontal chest X-rays of adult patients to support the assessment of endotracheal tube placement.	Tubus Position
eCARTv5 Clinical Deterioration Suite (eCART)	K233253	2024	FDA Database	✓	II			Analyzes EHR data from hospitalized adult ward patients to provide automated risk stratification and early warning of clinical deterioration	Clinical Deterioration
EFAI Chestsuite XR Malpositioned ETT Assessment System (ETT-XR-100)	K242821	2025	FDA Database	✓	II			Analyzes chest X-rays of adult patients to support the assessment of endotracheal tube placement.	Tubus Position
Global Hypoperfusion Index (GHI) Algorithm	K231038	2023	FDA Database	✓	II			Analyzes hemodynamic data from Swan-Ganz catheter monitoring to predict the likelihood of a global hypoperfusion event in surgical and non-surgical patients.	Clinical Deterioration
LOGIQ Totus	K232381	2023	FDA Database	✓	II	✓	IIa (MDR)^c^	Provides general-purpose diagnostic ultrasound imaging and analysis across a wide range of clinical applications for use by trained healthcare professionals in hospital and clinical settings.	Multiple
Lumify Diagnostic Ultrasound System	K223771	2023	FDA Database	✓	II			Provides portable diagnostic ultrasound imaging and fluid flow analysis across multiple clinical applications for use by trained healthcare professionals at the point of care.	Multiple
Lung AI (LAI001)	K243239	2025	FDA Database	✓	II			Analyses lung ultrasound scans of adults to flag regions suggestive of pleural effusion or consolidation/atelectasis.	Lung Pathologies
Migo	UDI 05700002261618	2025	Websearch			✓	IIa (MDR)	Provides continuous camera-based AI monitoring with real-time movement detection and notifications.	Multiple
MonaOS	UDI 04260735730047	2023	Websearch			✓	IIa (MDR)	Provides an AI-powered bedside assistant for intensive care that supports clinical decision-making, documentation, data management, and telemedicine.	Administration
NAVOY	not identifiable	2021	Persson et al.^9^			✓	not identifiable	Forecasts sepsis risk in ICU patients up to 3 h earlier than rule-based scoring systems by analyzing vital signs, laboratory data, and clinical information.	Sepsis
Pacmed Critical	UDI 08720908230089	2024	Websearch			✓	IIa (MDR)	Predicts the risk of death or readmission within 7 days of ICU discharge.	Post-discharge Deterioration
PeraMobile and PeraWatch System	K183370	2019	FDA Database	✓	II			Displays and trends Rothman Index scores with configurable alerts to support clinicians in monitoring patient status.	Multiple
PeraServe and PeraTrend System	K172959	2018	FDA Database	✓	II			Calculates and displays Rothman Index scores from EHR data to provide healthcare professionals with a patient status index for pediatric and adult populations as an adjunct to vital signs monitoring.	Multiple
PMcardio	UDI 04260738430135	2022	Websearch	^b^		✓	IIb (MDR)	Provides AI-powered ECG interpretation via mobile and institutional platforms to enable the detection of acute myocardial infarction and multiple cardiovascular diseases.	Cardiovascular Conditions
PMD-200	DEN210022	2021	FDA Database	✓	II	✓	not identifiable	Monitors nociception in anesthetized adult patients.	Sedation
RhythmAnalytics	K182344	2019	Lee et al.^8^	✓	II			Analyzes single-lead ECG recordings in adults to detect arrhythmias and ECG parameters	Cardiovascular Conditions
Sepsis ImmunoScore	DEN230036	2024	FDA Database	✓	II			Uses electronic health record data to assess adults in the hospital or the emergency department for risk of developing sepsis within 24 h.	Sepsis
Swoop Portable MR Imaging System	K251276	2025	FDA Database	✓	II	✓	IIa (MDR)	Mobile AI-enabled ultra-low field MRI scanner.	Multiple
Swoop Portable MR Imaging System (V2)	K250236	2025	FDA Database	✓	II			Mobile AI-enabled ultra-low field MRI scanner.	Multiple
T3 Platform software	K223578	2023	Websearch	✓	II	✓	IIa (MDR)	Records and displays physiologic and laboratory data from bedside devices and calculates risk indices to support clinicians in assessing oxygen delivery, ventilation, acid-base balance, and lactate status in pediatric and neonatal intensive care patients.	Oxygen Delivery Monitoring
TriVerity	K241676	2025	FDA Database	✓	II			Provides an automated blood test to differentiate bacterial and viral infections and assess risk of severe illness requiring organ support within 7 days in adults with suspected infection or sepsis.	Sepsis
U-Care Renal Platform	UDI G459UCR0	2023	Websearch			✓	IIb (MDR)	Provides AI-based predictive monitoring and management of acute kidney injury in ICU patients.	Kidney injury
Visensia	K081140	2008	FDA Database	✓	II	✓	IIa (MDD)	Combines multiple vital signs into a single index to alert healthcare professionals to changes in the physiological status of adult patients.	Multiple
WAVE Clinical Platform	K171056	2018	FDA Database	✓	II			Provides hospital clinicians with remote access to physiologic data, waveforms, alarms, and results from medical devices and information systems.	Multiple
x-cardiac-platform	UDI 04260752980012	2024	Websearch			✓	IIa (MDR)	Predicts postoperative bleeding and acute kidney injury after cardiac surgery.	Postoperative Bleeding

In addition to regulatory data, the table lists the source wfrom which the device was identified, provides a summary of the intended use of the device, and describes the disease or condition the device is intended for.

^a^Date not identifiable, the creation date of the unique device identifier (UDI) was provided.

^b^FDA Breakthrough Device Designation (not formally approved).

^c^Not identifiable on the website of the manufacturer, instead, the entry in the Slovakian State Institute for Drug Control (SUKL) database was used^34^.

In the US, the regulatory pathway for the majority of devices (*n* = 24) was the Premarket Notification 510(k) pathway, in which regulatory clearance is based on substantially equivalent devices (predicate devices). The other three US devices have been approved through a De Novo request. Among the 24 510(k) cleared devices, four name the CLEWICU System (ClewICUServer and ClewICUnitor) (K200717) as one of their predicate devices, two the PeraServe and PeraTrend System (K172959), two the Acumen Hypotension Prediction Index (HPI) Feature Software (DEN160044, not included in the study as not ICU specific), two the HemoSphere Advanced Monitor, HemoSphere ClearSight Module, Acumen Hypotension Prediction Index (K203687, not included in the study), and two the Swoop Portable MR Imaging System (K240944, not included in the study as not ICU specific). Typical small differences between the analyzed devices and their predicates include the use of different clinical indices and AI models, different target populations (e.g., exclusion of pediatric patients), changes in input data (e.g., inclusion of electronic health records (EHR)), or improved AI algorithms (e.g., for MRI image reconstruction). More substantial differences could be observed among the medical devices BriefCase, Critical Care Suite with Endotracheal Tube Positioning AI algorithm, and Lung AI (LAI001), where the predicate devices were intended for different conditions, anatomical structures, and image modalities.

**Fig. 2:**
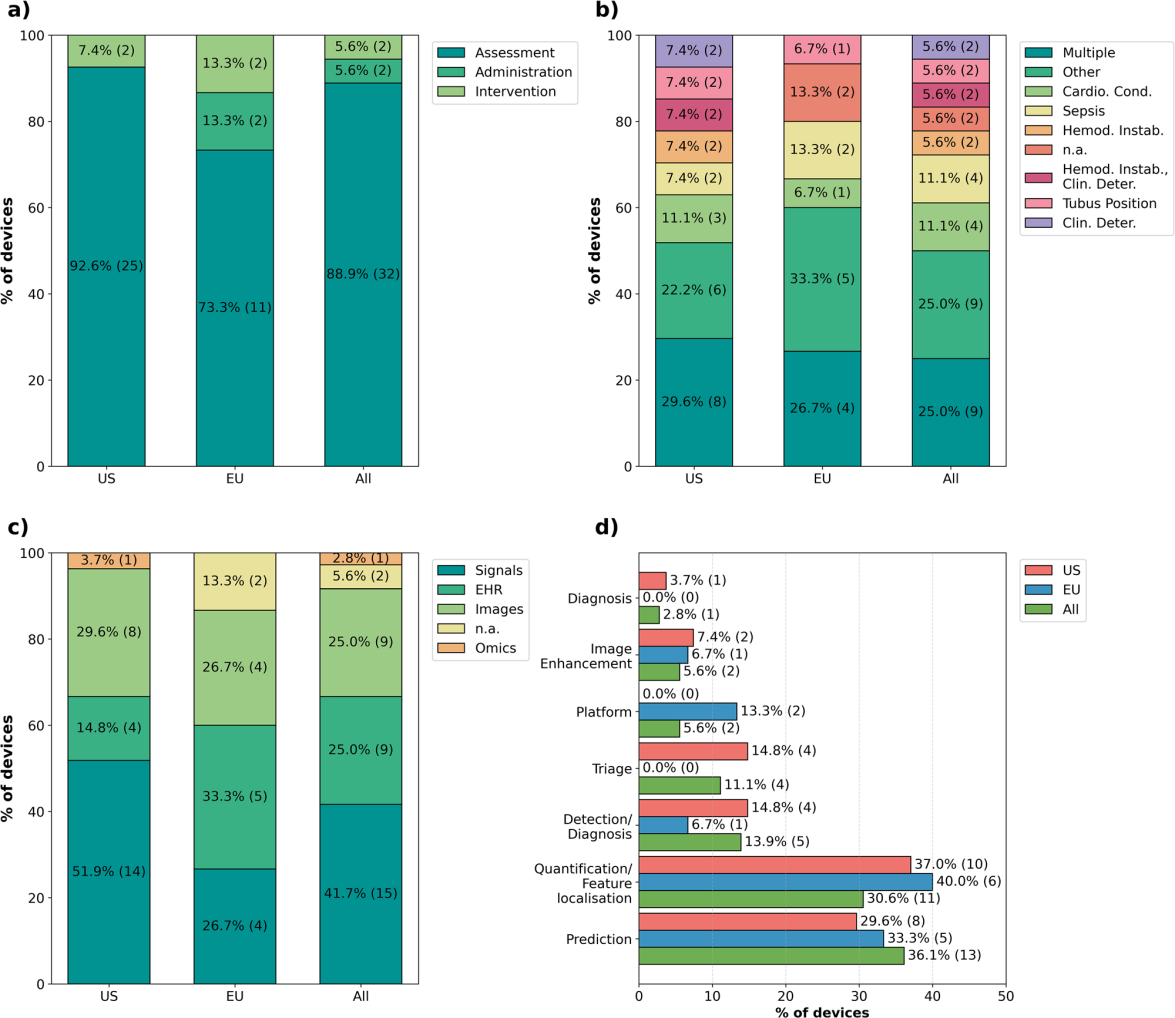
Clinical and technical aspects of AI devices intended for ICU use. **a** Clinical function, **b** intended disease/condition (Top 8), **c** data type used, **d** AI subfunction (some devices have more than one primary function).

Following the taxonomy established by Singh et al.^13^, the majority of ICU-AIeMD (*n* = 32, 88.9%) primarily have assessment functions (Fig. 2a). Most are intended for multiple clinical conditions, including the identification of various pathologies in ultrasound images (*n* = 9, 25.0%), followed by cardiovascular conditions (*n* = 4, 11.1%) and sepsis (*n* = 4, 11.1%) (Fig. 2b). Most systems rely on physiological signals such as vital signs or ECG signals (*n* = 15, 41.7%), followed by EHRs (*n* = 9, 25.0%), and imaging-based applications (*n* = 9, 25.0%). Two devices (5.6%) require no specific data input, as they primarily function as administrative platforms that could integrate other clinical AI solutions (Fig. 2c). The majority of devices focus either on quantification and feature localization-related tasks (*n* = 11, 30.6%), or on prediction (*n* = 13, 36.1%) (Fig. 2d). A smaller subset of devices is intended for detection and diagnostic purposes (*n* = 5, 13.9%), triage (*n* = 4, 11.1%), image enhancement (*n* = 2, 5.6%), and functioning as platforms or orchestration layers for other AI solutions (*n* = 2, 5.6%). The complete dataset including all functions is provided in the Supplementary Data.

Compared to the EU, the US market has a higher number of approved ICU-AIeMD, with 27 in the US and 15 in the EU (devices available in both regions are included in both the EU and US counts), as shown in both current and total authorizations (Fig. 1). Since 2021, both regions have shown sustained authorizations with year-to-year variability, including periods when the EU matched US authorizations (2022 and 2023). Additionally, the regulatory risk profiles of devices differ. This, however, stems from differences in the characteristics of authorized devices, not from different risk assessments, as all devices authorized in both regions have a similar risk class (II in the US, IIa in the EU). In both regions, the majority of devices are intended for assessment functions, while devices with an administrative clinical function are limited to the EU (Fig. 2a). US-cleared devices more frequently rely on physiological signals, whereas EU-certified devices more often incorporate EHR data (Fig. 2c). US devices have a higher relative presence of triage- and detection/diagnosis-focused applications, while a higher percentage of EU devices includes platform functions as well as quantification and prediction (Fig. 2d).

**Fig. 3:**
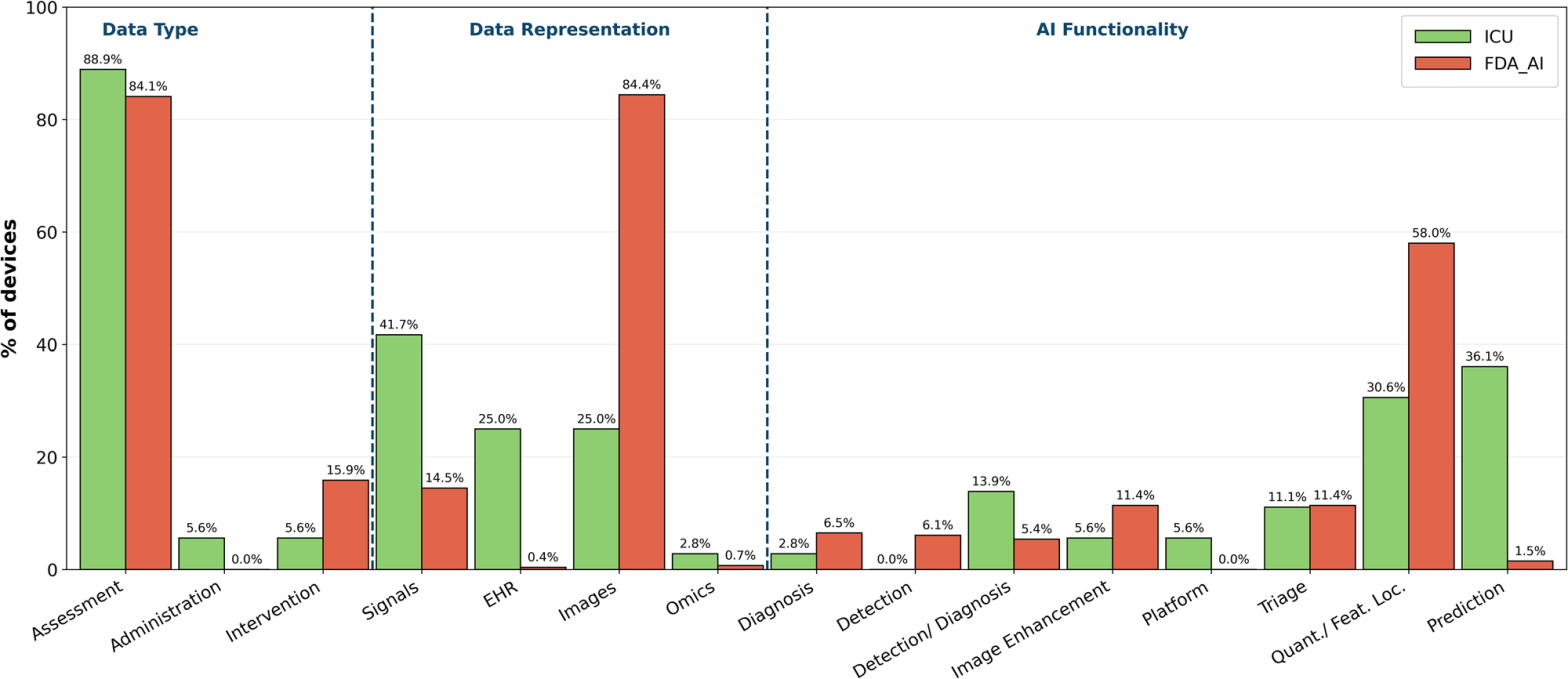
Comparison with the broader landscape of AI-enabled medical devices. The ICU-specific dataset was compared with the broader FDA data on AIeMDs provided by Singh et al.^13^ covering the metrics ‘Clinical function’, ‘Data type used’, and ‘Main AI function’. The variables ‘Administration’ (clinical function) and ‘Platform’ (AI subfunction) were not present in the FDA dataset and have been added for ICU-specific devices.

The comparison of ICU-AIeMD with the broader landscape of FDA-authorized AIeMDs, as described by Singh et al.^13^, reveals further distinctions. Across both datasets, most devices focus on assessment rather than intervention (88.9% in the ICU dataset, 84.1% in the general dataset). In the FDA-wide dataset, devices primarily depend on imaging (84.4%), while signals (14.5%), EHR/tabular data (0.4%), and omics (0.7%) are comparatively less represented^13^. In contrast, only 25.0% of ICU devices are imaging-based, while physiological signals (41.7%) and EHR data (25.0%) are overrepresented (Fig. 3). Singh et al. report that quantification/feature localization dominates the FDA-wide landscape (58.0% of all devices), followed by triage (11.4%), and image enhancement (11.4%), diagnosis (6.4%), detection (6.1%), and detection/diagnosis (5.4%), while predictive devices make up only 1.5% of all devices. By comparison, prediction (36.1%) and detection/diagnosis (13.9%) are substantially more prominent among ICU devices, while quantification/feature localization (30.6%) and image enhancement (5.6%) are underrepresented (Fig. 3).

In our study, we identified several ICU-AIeMDs already available in the US and EU. These findings challenge the view that the translation from AI research to practical devices has made little progress in intensive care medicine, as recently reported by Berkhout et al.^7^. A potential reason for this divergence might be that the state of implementation cannot be determined solely from clinical studies, as medical devices generally do not require peer-reviewed publications to be authorized and adopted^14^. Additionally, while a device can obtain regulatory clearance and enter the market with a limited publication footprint, many published models may never progress to a marketed product as they are developed without implementation in mind or are used only locally (in-house), which does not require regulatory authorization, either in the US or in Europe. On the other hand, the mere existence of devices and studies does not necessarily imply clinical implementation or widespread use. Researchers have described ICU-specific factors that contribute to the slow uptake of AI technologies, even when devices are already available, including the high criticality of patients, the necessity of fast decision-making, a complex environment with multiple data sources, and high patient variability^15^. Additional challenges include that algorithms are usually developed retrospectively on historical data, that training data may differ from real-world data (with this problem worsening over time through model drift)^3,16^, a lack of model interpretability and transparency^15,17^, automation bias^18^, and alarm fatigue^3,16,19^. ICU environments already suffer from high alarm burden^20^, and AI-enabled monitoring systems risk amplifying this challenge unless they demonstrate not only sensitivity but also specificity and contextual prioritization. Beyond site-level adoption, transporting ICU AI models between institutions remains difficult due to diverse documentation practices and fragmented medical-device and IT ecosystems^21^.

Additionally, regulatory uncertainty, especially regarding adaptive and continuously learning algorithms, should be considered as an implementation barrier, as current authorization frameworks remain predisposed to “locked” algorithms, systems fixed at the time of authorization, whereas many modern AI approaches are adaptive, learning continuously as new data are received^22–24^. Regulators can validate the version submitted for authorization, but cannot guarantee that a continuously learning system will remain safe and effective in future states. This limitation is particularly concerning in ICUs, where even slight shifts in model performance may lead to clinically significant outcomes.

In critical care research, AI is often described as a tool to support continuous monitoring, early warning, prognosis, complication prediction, and the optimization of supportive therapy, rather than as a predominantly image-interpretation technology^2,3,25–27^. While in early development stages, prediction of mortality, prognosis, and complications account for the largest share of models ( > 74% according to Berkhout et al.^7^), more mature applications are dominated by video and image assessment and the improvement of mechanical ventilation^7^. Both aspects are somewhat reflected in our results. Image-based devices and devices intended for quantification/feature localization make up a substantial share, but predictive devices surpass them. Although most ICU-focused devices are categorized as assessment tools, this classification obscures an important clinical distinction: whether outputs merely add information or actively shape downstream decisions. Predictive scores and alerts influence care only when they are embedded in escalation pathways (e.g., intensified monitoring, early diagnostics, or therapy initiation). Without such coupling, prediction risks remain advisory rather than actionable, regardless of regulatory status. Thus, future AI systems should be evaluated not only for predictive accuracy but also for their net effect on ‘signal-to-noise ratio,’ usability, cognitive load, and fit to clinical workflows, and, ideally, measurable net positive outcome effects under real-life implementation conditions.

While Singh et al. provide a specialty-agnostic mapping of all FDA-authorized AIeMDs, our analysis adds a domain-specific and cross-jurisdictional perspective, characterizes only devices intended for ICU use, provides deeper insight into their clinical functions, and, to our knowledge, is the first structured identification of such devices in the EU. When considered within this broader AIeMD landscape, ICU-specific devices seem to form a distinct category characterized by different data types and use cases. In Singh et al.’s FDA-wide analysis, imaging predominantly serves as the primary input modality, and quantification and feature localization are the most common AI functions^13^, aligning with the fact that most devices are developed for radiology and other imaging-focused specialties^28^. Our findings show that ICU devices differ substantially. This divergence, however, makes sense when the clinical context of use is considered: many ICU-relevant problems are time-dependent and time-critical and benefit from models that prioritize temporal risk estimation, early detection, and trend-based monitoring^3,29^, and are able to ingest the high frequency physiological measurement data typical for ICU settings, whereas imaging specialties more often support episodic interpretation tasks in comparatively standardized acquisition settings^30^. The prominence of prediction among ICU devices may also indicate where translation has been most feasible. Prediction and early warning systems align directly with recognized ICU potential and needs^29^. At the same time, the on-market focus on relatively narrow prediction and quantification tasks highlights that current regulated products still cover only part of the functionality spectrum described in ICU AI research, particularly compared with more integrated, agentic workflow-orchestrating systems, generative AI systems, or broader adaptive approaches^5,7^.

Cross-regional differences primarily occur in authorization numbers, risk classifications, and characteristics. The US usually shows a higher annual authorization rate and an overall higher absolute number. This difference, however, mainly results from a temporal offset rather than fundamentally different growth dynamics. After the first authorization of Visensia in 2008 in the US and 2010 in the EU, authorization resumed in the US in 2018, whereas the first comparable EU authorization occurred in 2021. Beyond counts, ICU-focused medical devices differed in regulatory profiles between regions. These differences in classification have been described in other health applications^31^. However, in our ICU dataset, this difference stems from differences in the characteristics of approved devices, not from different risk assessments, as all devices approved in both regions have a similar risk class (II in the US, IIa in the EU). An additional observation is that administrative and platform-like products are generally uncommon in the US, whereas two such products have been identified in the EU. These findings may reflect regulatory differences, where purely administrative tools as well as many clinical decision support applications are not classified as medical devices in the US, or differences in development practices, where orchestrative systems are developed internally (in-house development) and thus do not appear as commercial, certified products. Additionally, they might indicate regulatory uncertainty in the EU, where some developers seek regulatory certification (e.g., aiOS), while others do not (e.g., deepcOS). A similar divergence in regulatory approaches can be seen in CDSS, which might be classified as a non-device if they follow the boundaries outlined in FDA guidance, including if such systems are solely intended for physicians, do not acquire or process images, and only offer recommendations without overriding clinical judgment^32^. Another US-specific aspect to consider is the predominant clearance of devices through the 510(k) pathway. The findings emphasize that cross-regional comparisons should be considered in light of structural regulatory differences, since these may influence the comparability of device landscapes and the availability of data.

Our findings point to several priorities for the field. Importantly, regulatory clearance or certification of ICU-AIeMDs should not be conflated with proven patient benefit. Demonstrating outcome improvements will require prospective evaluation in real-world settings, careful workflow integration, and systematic monitoring of AI-specific risks that may not be fully captured by current regulatory processes. Accordingly, pragmatic clinical trials, implementation-focused study designs, and robust post-market surveillance strategies that link device use to clinically meaningful ICU outcomes and safety endpoints should be prioritized^33^. In parallel, regulatory pathways for generative AI and agentic systems remain an important open question for the next generation of critical care AI, and future work should examine how existing frameworks can accommodate highly adaptive or multi-purpose models. Finally, a more comprehensive analysis of AI-enabled medical devices in the European market is necessary, one that is not restricted to a single clinical area.

In conclusion, our analysis indicates that ICU-AIeMDs are already available in both the US and EU markets, with a stronger emphasis on prediction functions and signal/EHR-based inputs than in the broader AIeMD landscape. Currently, the EU has fewer ICU-focused devices identified compared to the US, with notable differences in risk-class distributions and technical and functional profiles, though comparability is limited due to lower EU regulatory transparency. Our findings help to partly resolve the ambiguity between a research-focused perspective on ICU AI and the existence of regulated products. However, availability does not equal proven benefit, and the current device landscape remains dominated by narrow, task-specific tools. Wider adoption will probably depend less on the availability of additional single-task models and more on addressing persistent implementation barriers, embedding predictive outputs into actionable clinical pathways, generating prospective outcome evidence, and developing regulatory approaches suited to adaptive and generative AI systems.

## Methods

To describe the current landscape of approved medical devices intended for use in ICU settings, we sought to identify approved and commercially available AIeMDs with ICU-relevant intended purposes.

### FDA database search

For the US search, the device summaries of all medical devices listed in the FDA’s list of Artificial Intelligence-Enabled Medical Devices (https://www.fda.gov/medical-devices/software-medical-device-samd/artificial-intelligence-enabled-medical-devices) were downloaded on July 21st, 2025. A systematic text search was applied to device summaries for ICU-related terms (e.g., “intensive care,” “critical care,” “hemodynamic instability,” “ventilation,” “sepsis”) using Python v3.11.6 with the extension *pdfplumber*, and results were validated by manual review. A complete set of keywords is provided in the Supplementary Material, Supplementary Table 1. To identify any devices that were approved between the last update of the AI-enabled devices list and the cut-off date for this study, the product summaries of all devices approved between 10th July 2025 and 21st July 2025 have been downloaded and searched for AI-related (‘artificial intelligence’, ‘deep learning’, ‘machine learning’, ‘software’, ‘AI’, ‘ML’) as well as the ICU-related terms mentioned earlier. When summaries could not be automatically processed, a human reviewer screened them directly. Devices were included if the intended use specified the use of AI, the use in ICU settings, or ICU-relevant diseases. Metadata, including year of authorization, regulatory pathway, risk class, predicate devices (for 510(k)-cleared devices only), and intended use statement, was extracted.

### EU search

A direct query of the official medical device registry in the European Database on Medical Devices (EUDAMED) was not possible, as this database does not contain comprehensive information about devices, summaries, or intended use statements. Considering these limitations, we conducted a multimethod structured search. All devices identified in this search were added to a database.

#### US database cross-referencing

Every device intended for the US market was cross-checked against the EUDAMED database on July 22nd 2025. Given the potential variability in device names, the manufacturer’s names were also searched within EUDAMED to verify correspondence with those approved in the US. Devices that could not be identified in EUDAMED underwent manual searches, including reviews of the manufacturer’s website and supplementary web searches utilizing the device’s name, the manufacturer’s name, and keywords such as “CE” or “MDR.”

#### Literature and web search

To identify devices that are only present on the European market, the scientific literature database PubMed was searched on June 16th, 2025 using the following search term: (“artificial intelligence”[Title/Abstract] OR “machine learning”[Title/Abstract] OR “deep learning”[Title/Abstract] OR “AI”[Title/Abstract]) AND (“intensive care”[Title/Abstract] OR “critical care”[Title/Abstract] OR “ICU”[Title/Abstract] OR “intensive care units”[MeSH Terms] OR “critical care”[MeSH Terms]) AND (“Medical Device”[Title/Abstract] OR “product”[Title/Abstract]). The devices described in the 29 identified studies were cross-referenced with the US FDA device database and EUDAMED to verify their existence and added to the project database. In a second step, a structured web search was conducted on June 16th, 2025, using the advanced search feature of the Google search engine in a de-personalized browser. The following search term was used: (“Artificial Intelligence” OR “Machine Learning” OR “Deep Learning” OR “Neural Network” OR “Computer Vision” OR “Clinical Decision Support” OR “AI-based” OR AI) AND (“CE marked” OR “CE marking” OR MDR OR MDD OR “Notified Body” OR “Approved”) AND (“Intensive Care” OR “Critical Care” OR ICU OR NICU OR PICU OR “Emergency Care” OR Ventilation OR Hemodynamic OR “Patient Monitoring” OR Sepsis OR ARDS OR “septic shock” OR “acute respiratory distress syndrome” OR “multi-organ failure” OR “cardiac arrest”), which led to 166 results. All identified websites have been assessed, and the mentioned devices have been extracted. Results were manually reviewed, and inclusion was based on regulatory authorization status and on either an explicit reference to ICU use or clear applicability in ICU-relevant tasks. Additional information about these devices has been obtained through publicly available regulatory documents, manufacturer websites, and academic publications.

#### Identification of the intended purpose for EU devices

Since the intended use statement of EU-only devices is not publicly available, the manufacturer’s website was searched for a summary or description of the device. The metadata (year of certification, risk class, UDI, manufacturer) was extracted from EUDAMED, if possible. If not, the manufacturer’s website was searched for press releases, statements, and certificates. An additional web search was conducted if the manufacturer’s website did not contain any information, including the Slovakian State Institute for Drug Control (SUKL) medical device database^34^. Devices were included if the intended use specified the use of AI, the use in ICU settings, or ICU-relevant diseases. All devices identified in this search were added to a database.

### Device categorization

The included devices were categorized in accordance with the taxonomy proposed by Singh et al.^13^ to allow comparison with the broader landscape of FDA-approved devices. In this framework, each device is categorized along three axes: clinical function, AI function, and data type. Clinical functions include Assessment (e.g., diagnosis and monitoring) and Intervention (e.g., treatment guidance and surgery). AI function is categorized into two main categories: Analysis and Generation. Analysis refers to devices in which AI is applied to interpret or evaluate existing data, whereas Generation refers to devices in which AI is involved in producing or optimizing the underlying data itself, for instance, through reconstruction, enhancement, or synthesis^13^. Accordingly, an imaging system that acquires data without AI but includes an AI module for interpretation would be classified as Analysis, whereas one that uses AI for image reconstruction is classified as Generation^13^. The AI functionality is then further subdivided into subclasses. Within Analysis, six subclasses are defined: triage (a binary alert to prioritize review), quantification/feature localization (deriving quantitative metrics and/or positional information, often together, e.g., segmentation followed by volumetry), detection (helping to identify disease-suspicious regions or time intervals), diagnosis (a case-level score or category estimating the likelihood of a specific diagnosis), detection/diagnosis (devices that explicitly perform both, kept as a separate class), and prediction (estimating future risk rather than current status). Within Generation, three subclasses are defined: image enhancement (e.g., denoising or AI-based reconstruction), acquisition guidance (assisting in proper data capture or positioning without creating the primary data used downstream), and synthetic data generation (producing new data, such as synthesizing a CT image from MRI or an ECG lead from existing leads). Data types are assigned by the core input to the AI model and include images, physiological signals, electronic health records, and omics. A comprehensive description of the taxonomy is provided in Singh et al.^13^.

**Fig. 4:**
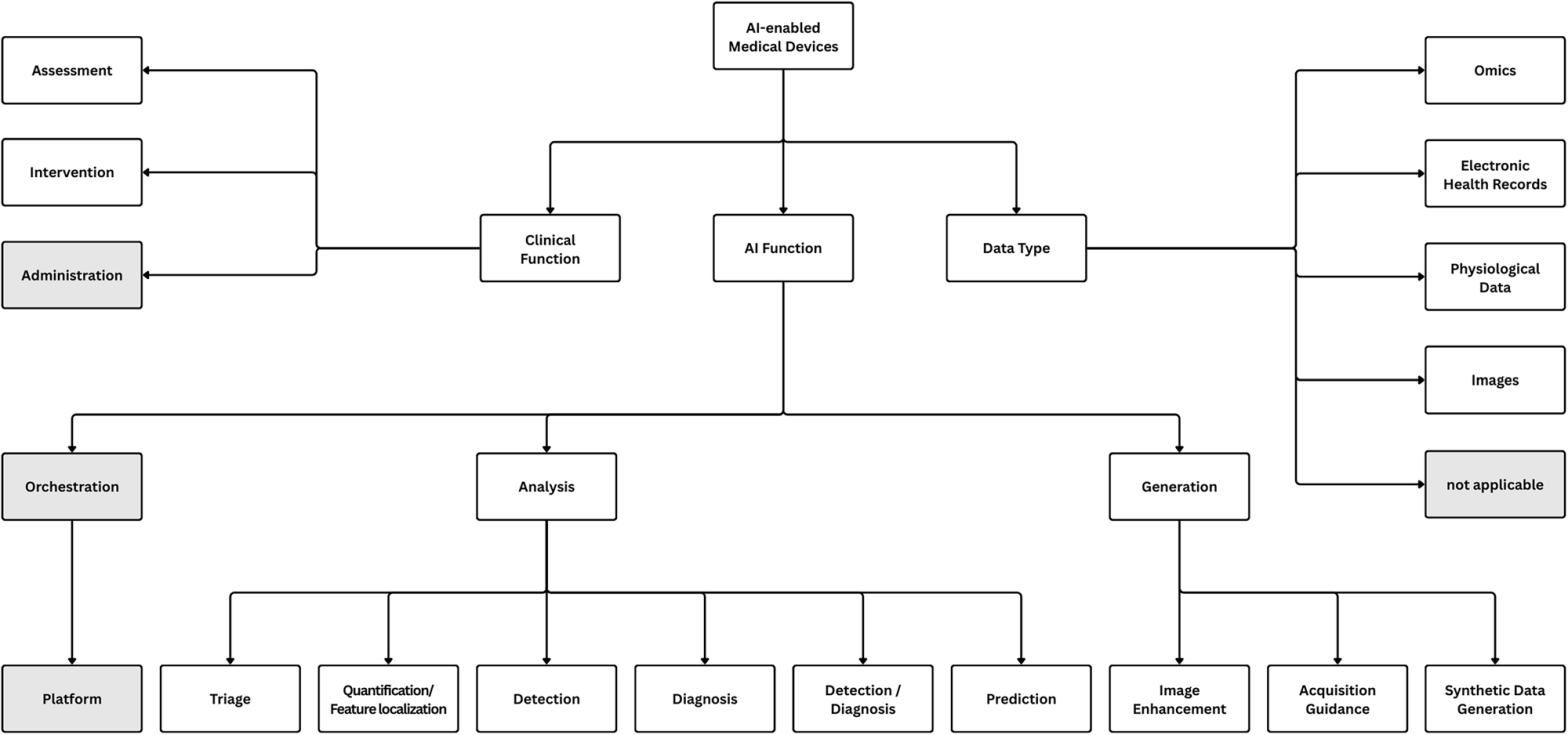
Flowchart of the device categorization process. Gray boxes indicate categories that have been added to Singh et al.’s taxonomy^13^.

Since not all devices identified in our search could be categorized along these functions, we introduced three extensions to the taxonomy. First, we added a third clinical function, Administrative, defined as devices whose primary purpose is organizational or operational (e.g., resource management or workflow coordination) rather than direct patient assessment or intervention. Second, we added a third primary AI function, Orchestration, defined as devices in which AI is used to integrate, coordinate, or manage other algorithms or data streams rather than performing analysis or generation itself. Third, we added a corresponding AI subfunction, Platform, for systems that host or provide an infrastructure layer to integrate other AI-enabled clinical applications. These extensions were necessary because two EU-certified devices did not fit the existing categories of Assessment/Intervention and Analysis/Generation established by Singh et al. A flowchart of the categorization process is shown in Fig. 4, and a Sankey diagram is provided in the Supplementary Material, Supplementary Fig. 1.

The categorization was conducted based on the intended use statement of the device (for devices approved in the US) or the description or summary provided on the manufacturer’s website (for devices approved in Europe). Additionally, the primary target condition or disease was identified. The extraction and classification were conducted by a human researcher with a medical degree (author OF) and reviewed by the other authors.

### Data analysis

Descriptive analyses were conducted using Python (v3.11.6). Figures were generated in Python to illustrate temporal authorization trends, regulatory classifications, and clinical/AI functions. ICU-specific devices were compared across the US and EU. To contextualize the ICU-specific findings, ICU-specific devices (all 36) were compared with the broader FDA-approved device landscape described by Singh et al.^13^. As no similarly comprehensive, device-level dataset was available for the EU at the time of writing, no sub-analysis for the EU could be conducted.

### Limitations

This study has some limitations. First, while identifying AIeMD in the US is possible via the Devices@FDA database, no system with directly comparable functionality exists for the EU. Although we developed a systematic and detailed search protocol, it remains possible that some devices on the EU market may not have been identified in this study. Second, the FDA database categorizes devices into broad clinical specialty groups without a specific category for ICU-specific devices. Consequently, the identification of US-approved ICU-specific products could be incomplete. Third, this study focused on devices explicitly intended for ICU settings or critical care. Thus, devices with a broad range of purposes that did not state such target areas explicitly have been excluded, as well as non-commercial in-house developments. Additionally, the findings are not necessarily generalizable to all specialties. Fourth, the FDA’s Artificial Intelligence-enabled Medical Devices database might itself be incomplete.

## Supplementary information

Supplementary Information

Supplementary Information

## Data Availability

All data and materials generated and analyzed during this study are included in the article and in the data supplement.
