# Supplementary material for: The landscape of artificial intelligence-enabled medical devices in the EU and the US intended for intensive care units
Source: NPJ Digit Med. 2026 Apr 10;9:322. doi: 10.1038/s41746-026-02609-2 (PMC13087237; doi:10.1038/s41746-026-02609-2)
Supplement: Supplementary file 1 — Supplementary Information [file 41746_2026_2609_MOESM1_ESM.pdf]

The landscape of Artificial Intelligence-enabled Medical Devices in the EU and the US intended for Intensive Care Units - Supplementary Material

Oscar Freyer[1¶], Stephan Buch[1], Adel Bassily-Marcus[2], Sven Zenker[3], Brian W. Pickering[4], Max Ostermann[1], Anett Schönfelder[1], Stephen Gilbert[1]

1 - Else Kröner Fresenius Center for Digital Health, TUD Dresden University of Technology, Dresden, Germany

2 - Yale School of Medicine, Department of Surgery, Yale New Haven Health System, New Haven, United States of America

3- Staff Unit for Medical & Scientific Technology Development & Coordination (MWTek); Department of Anesthesiology and Intensive Care Medicine; Institute for Medical Biometry, Informatics, and Epidemiology, University Hospital Bonn, Bonn, Germany

4- Department of Anesthesiology and Perioperative Medicine, Mayo Clinic, Rochester, United States of America

¶- corresponding author: Dr. Oscar Freyer, oscar.freyer@tu-dresden.de, Fetschersr. 74, 01307 Dresden

Table of Contents

Regulatory Context 2

Supplementary Figures 3

Supplementary Figure 1. Sankey diagram displaying all included devices 3

Supplementary Tables 4

Supplementary Table 1. Keywords used in Searches. 4

Supplementary Table 2. Device Database. 5

References 6

Regulatory Context

Since many AI solutions have medical functionality (e.g., disease detection and prediction) and are therefore regulated as medical devices (at least when marketed commercially or used beyond their health institution of development), large-scale implementation typically requires regulatory authorization through a strict process defined in legislation. Applications without a medical purpose (e.g., generation of discharge letters) are usually not regulated as medical devices. For certain types of devices, such as clinical decision support systems (CDSS) in the US, the regulatory rules are more nuanced[1]. A similar divergence in regulatory approaches can be seen in CDSS, which might be classified as a non-device if they follow the boundaries outlined in FDA guidance, including if such systems are solely intended for physicians, do not acquire or process images, and only offer recommendations without overriding clinical judgment[2].

Both jurisdictions apply a risk-based system, where devices could fall into one of multiple risk classes, depending on their intended use, characteristics, and the general risk they pose to patients. A higher risk class often leads to more regulatory expectations. While the US uses three risk classes, Class I (low risk), Class II (medium risk), and Class III (high risk), the EU legislation under the Medical Device Regulation (MDR) primarily uses four classes: Class I (low risk), Class IIa (medium-low risk), IIb (medium-high risk), and Class III (high risk). While regulatory authorization via US Food and Drug Administration (FDA) approval or clearance or European Conformité Europénne (CE) marking is a prerequisite for commercialization, these certifications primarily establish that a device meets standards of safety and technical performance, and in practice do not consistently demonstrate clinical effectiveness, even though the proof of clinical benefits is foundational to the European process. For clinicians, this distinction can be unclear: authorization may create the perception that an AI tool has proven impact on patient outcomes, when in fact many devices reach the market without prospective trials confirming reductions in morbidity, mortality, length of stay, or other clinically relevant outcomes or outcome surrogates, or at least without information on such studies being available on the public record. This gap between regulatory authorization and proof of clinical utility highlights the need for targeted evaluation studies and careful integration into ICU workflows before widespread adoption[3].

While regulatory authorization via US Food and Drug Administration (FDA) approval or clearance or European Conformité Europénne (CE) marking is a prerequisite for commercialization, these certifications primarily establish that a device meets standards of safety and technical performance, and in practice do not consistently demonstrate clinical effectiveness, even though the proof of clinical benefits is foundational to the European process.

Supplementary Figures

Supplementary Figure 1. Sankey diagram displaying all included devices. The first division indicates the data type, the second shows the clinical function, and the third presents the AI function.

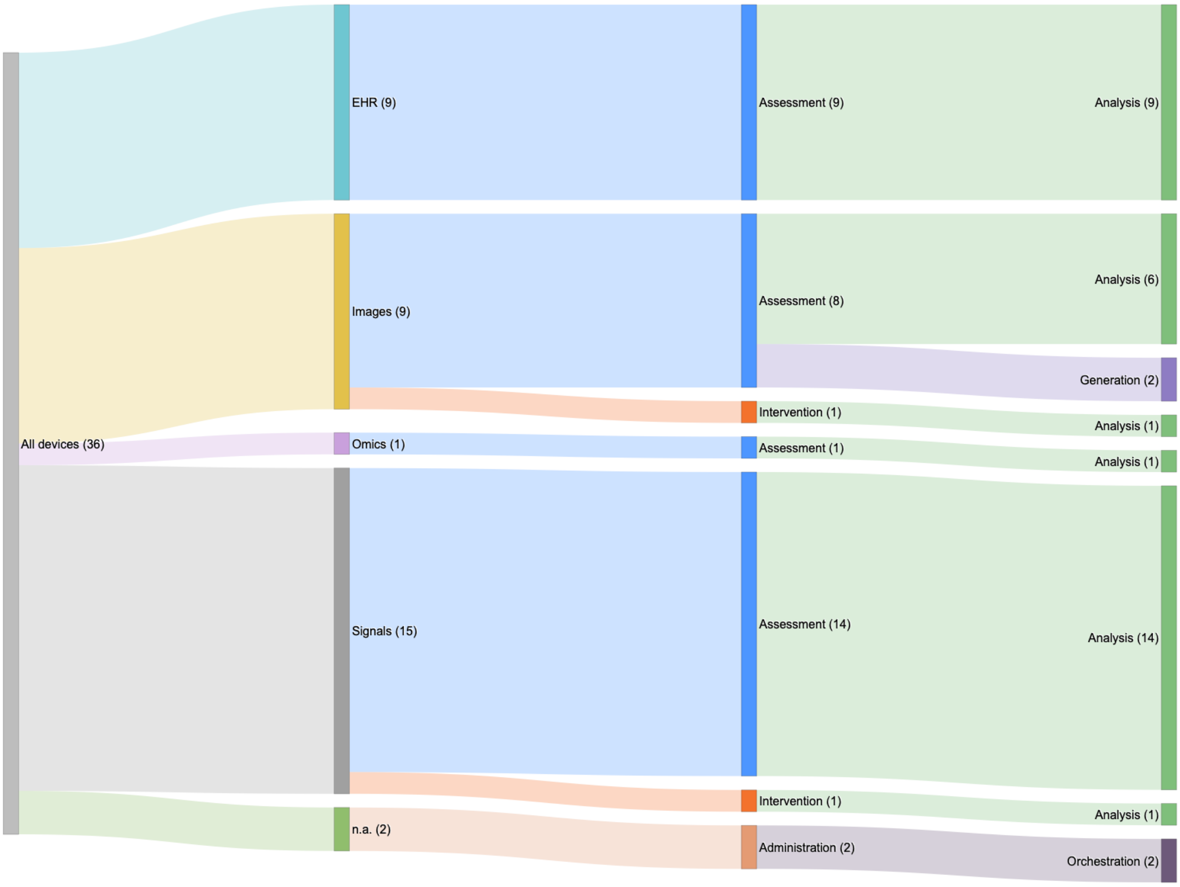

Supplementary Tables

Supplementary Table 1. Keywords used in Searches.

Search	Keywords
AI Devices	'artificial intelligence'<br>'deep learning'<br>'machine learning'<br>'software'<br>' AI '
ICU Devices	'intensive care unit'<br>'icu'<br>'critical care unit'<br>'ccu'<br>'emergency department'<br>'ed'<br>'trauma center'<br>'sepsis'<br>'septic shock'<br>'acute respiratory distress syndrome'<br>'ards'<br>'multi-organ failure'<br>'hemodynamic instability'<br>'cardiac arrest'<br>'sedation'<br>'anesthesia'<br>'anesthesiology'<br>'sedated'<br>'sedative'<br>'mechanical ventilation'<br>'vasopressor'<br>'extracorporeal membrane oxygenation'<br>'ecmo'<br>'intracranial pressure monitoring'<br>'life support'<br>'life-threatening'<br>'critically ill'<br>'respiratory failure'<br>'cardiogenic shock'<br>'hemorrhagic shock<br>'traumatic brain injury'<br>'tbi'<br>'acute kidney injury'<br>'aki'

	'coagulopathy'<br>'status epilepticus'<br>'arterial line'<br>'pulmonary artery catheter'<br>'swan-ganz'<br>'cardiac output monitoring'<br>'capnography'<br>'continuous eeg'<br>'intubation'<br>'resuscitation<br>'defibrillation'<br>'cardioversion'<br>'dialysis'<br>'intra-aortic balloon pump'<br>'iabp'<br>'inotropic support'<br>'inotrope'<br>'life-sustaining'<br>'acute care'<br>'emergency use'

Supplementary Table 2. Device Database.

The device database is provided as a separate Excel file titled "Supplementary Data." It includes the name, manufacturer, submission number/decision number/UDI, year of first authorization, the source from which the device was identified, whether it is present on the US market and the corresponding risk class, whether it is on the EU market and the corresponding risk class, a summary of the intended use/description (based on FDA summary and manufacturers' website), data type, clinical function, disease/conditions the device is intended for, main AI function (Analysis, Generation, Orchestration), and sub AI function (Quantification, Triage, Detection, Diagnosis, Detection/Diagnosis, Prediction, Image Enhancement, Acquisition Guidance, Synthetic Data Generation, Platform).

References

1. Gottlieb, S. New FDA policies could limit the full value of AI in medicine. *JAMA Health Forum* **6**, e250289 (2025).

2. U.S. Food and Drug Administration (FDA). Clinical Decision Support Software - Guidance for Industry and Food and Drug Administration Staff. Preprint at https://www.fda.gov/regulatory-information/search-fda-guidance-documents/clinical-decision-support-software (2022).

3. Kelly, C. J., Karthikesalingam, A., Suleyman, M., Corrado, G. & King, D. Key challenges for delivering clinical impact with artificial intelligence. *BMC Med.* **17**, 195 (2019).
